# Stereotactic body-radiotherapy boost dose of 18 Gy vs 21 Gy in combination with androgen-deprivation therapy and whole-pelvic radiotherapy for intermediate- or high-risk prostate cancer: a study protocol for a randomized controlled, pilot trial

**DOI:** 10.1186/s13063-018-2574-y

**Published:** 2018-04-02

**Authors:** Yeon Joo Kim, Hanjong Ahn, Choung-Soo Kim, Jae-Lyun Lee, Young Seok Kim

**Affiliations:** 1 Department of Radiation Oncology, Asan Medical Center, University of Ulsan, College of Medicine, 88, Olympic-ro 43-gil, Songpa-gu, Seoul, 05505 Republic of Korea; 2 Department of Urology, Asan Medical Center, University of Ulsan, College of Medicine, Seoul, Republic of Korea; 3 Department of Oncology, Asan Medical Center, University of Ulsan, College of Medicine, Seoul, Republic of Korea

**Keywords:** External-beam radiotherapy, Prostate cancer, Stereotactic radiotherapy boost

## Abstract

**Background:**

Combination therapy using external-beam radiotherapy (EBRT) with a brachytherapy boost has demonstrated superior biochemical control than dose-escalated EBRT alone. Whereas brachytherapy is disadvantageous because it is an invasive procedure, stereotactic body-radiotherapy (SBRT) using CyberKnife could emulate the dose distribution of brachytherapy and is a non-invasive and safe modality to control intra-fractional movement. We therefore adopted SBRT using CyberKnife as a boost therapy after whole-pelvic radiotherapy (WPRT).

**Methods/design:**

In this prospective, randomized, single-center, pilot study for intermediate- and high-risk prostate cancer without nodal or distant metastasis, after androgen-deprivation therapy and WPRT, patients will be randomized to one of two SBRT boost regimens, i.e., 18 or 21 Gy administered in three fractions every other day.

**Discussion:**

The aim of this trial is to evaluate acute toxicities using both physician- and patient-reported outcomes and short-term biochemical control with SBRT boost following WPRT. Additionally, chronic toxicities and long-term biochemical control will be evaluated as secondary endpoints in this trial. Based on the generated results, we will plan the full-scale phase II study for selecting the SBRT boost dose.

**Trial registration:**

ClinicalTrials.gov, ID; NCT03322020. Retrospectively registered on 26 October 2017.

**Electronic supplementary material:**

The online version of this article (10.1186/s13063-018-2574-y) contains supplementary material, which is available to authorized users.

## Background

High-dose external-beam radiotherapy (EBRT) with a prescription dose of 76–80 Gy is the current standard treatment for intermediate- and high-risk prostate cancer [1–3]. Recently, some studies have reported that combination therapy using EBRT with a brachytherapy boost demonstrated superior biochemical control than dose-escalated EBRT alone [4–6]. However, brachytherapy has several disadvantages including invasiveness, the need for anesthesia, insertion of multiple catheters, pain control, and hospitalization. Among the several approaches to replace brachytherapy boost with non-invasive EBRT boost techniques, stereotactic body-radiotherapy (SBRT) boost using the CyberKnife robotic radiosurgery system (Accuray Incorporated, Sunnyvale, CA, USA) is one of them. A previous trial reported that dose distribution with CyberKnife was comparable to that of brachytherapy [7]. One other advantage of CyberKnife over other SBRT delivery systems, such as volumetric modulated arc therapy, is intra-fraction motion control.

With this rationale in mind, whole-pelvic radiotherapy (WPRT) followed by SBRT boost using CyberKnife anticipates that biochemical control will be superior to the conventional high-dose EBRT. In addition, medical expenses will be lower because of a shorter total treatment time. Traditional fraction size has been 1.8–2 Gy; therefore, the duration of EBRT is 8–9 weeks. A longer EBRT is a heavy burden for both patients and the health care system. Because prostate cancer seems to have greater sensitivity to higher doses per fraction than that of organs nearby, [8] increasing the fraction size, called hypofractionation, has been adopted for prostate cancer [9, 10]. However, the implementation of hypofractionation with WPRT is challenging because of the concern of toxicity. However, administration of SBRT as boost therapy after WPRT can shorten the total treatment period to approximately 5 weeks. Several retrospective studies have assessed the clinical outcomes of WPRT with 45-Gy administered in 25 fractions, followed by a CyberKnife boost of 18–21 Gy in three fractions and demonstrated feasibility of the SBRT boost after WPRT [11, 12]. However, the boost dose and other modalities used in combination, such as androgen-deprivation therapy (ADT), varied among the patient population.

This prospective, randomized, single-center trial evaluates the acute toxicities and short-term control of prostate-specific antigen (PSA) of combination treatment including ADT, WPRT, and an SBRT boost. This trial uses two SBRT boost dose regimens, 18 and 21 Gy, both administered in three fractions. Based on the results of the study, we will design the full-scale phase II study for selecting the appropriate SBRT boost dose.

## Methods/design

### Recruitment and study design

This prospective, randomized, single-center, pilot study on intermediate- and high-risk prostate cancer patients is ongoing at Asan Medical Center. Patients are randomized into two groups with a 1:1 allocation using blocked randomization, and the random block size is 4. The randomization sequence is generated by the clinical research nurse using Excel software. The allocation sequence is concealed from the primary investigator who enrolls the patients using the Excel file locked with a password. Also, the file is saved in a computer located in the place that the primary investigator cannot approach. One group will receive a CyberKnife SBRT boost dose of 18 Gy administered in three fractions (18-Gy group), and the other group will receive a CyberKnife SBRT boost dose of 21 Gy administered in three fractions (21-Gy group).

**Fig. 1:**
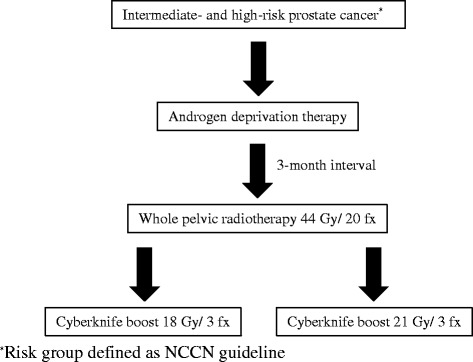
Flowchart of treatments for the trial. ^*^Risk group defined as per National Comprehensive Center Network (NCCN) guidelines

As shown in Fig. 1, all patients will first receive ADT. Several studies strongly support the use of ADT in addition to EBRT, long-term (i.e., 2–3 years) ADT for high-risk patients [13, 14] and short-term ADT (4–6 months) for the intermediate group [15, 16]. Risk group definition is based on the current National Comprehensive Center Network guidelines (https://www.nccn.org/professionals/physician_gls/pdf/prostate.pdf). Informed consent of patients is mandatory before recruitment and is obtained from the radiation oncologists. Three months after the start of ADT, WPRT (44 Gy in 20 fractions) with intensity-modulated radiotherapy (IMRT) will be initiated. Next, CyberKnife boost will be implemented. Since the primary investigator has to confirm the plan for CyberKnife boost, the blinding is not possible afterward.

The dose schedules are designed to achieve the biological effective dose (BED) as that of the conventional regimen with 78 Gy administered in 38 fractions. BED is widely used in radiation oncology to compare diverse radiation dose regimens using different fraction sizes, fraction numbers, and total doses [17]. For calculation of BED, an α/β ratio constant, i.e., the dose at which cellular death rates with linear and quadratic components are equivalent, is needed. A usual α/β ratio for normal tissue is 3, whereas that for prostate cancer is 1.5. As summarized in Table 1, the normal tissue BED for conventional regimen is 130.0 Gy, which is similar to that for the boost regimen of 18 Gy (130.7 Gy) and lower than that for a boost regimen of 21 Gy (146.7 Gy). The BED values for prostate cancer are 182.0, 197.3, and 226.3 Gy with conventional, 18-Gy boost, and 21-Gy boost therapies, respectively. As result, the 18-Gy group will receive the same BED for adjacent organs, while the tumor will receive 110% BED of conventional EBRT, while the 21-Gy group will be irradiated 110% and 120% BED of conventional EBRT for normal organ and prostate cancer, respectively.

**Table 1:** Biological effective dose (BED) calculations

	Conventional dose (78 Gy/28 fractions)	18-Gy group	21-Gy group
Normal tissue BED^a^ (Gy)	130.0	130.7	146.7
Prostate cancer BED^a^ (Gy)	182.0	197.3	226.3

^a^α/β ratio for normal tissue is 3, and 1.5 for prostate cancer

### Inclusion criteria

Patients fulfilling the following criteria are eligible for this trial: age, 20 years or older; diagnosis of pathologically confirmed intermediate- or high-risk prostate cancer within 6 months after enrollment; and an Eastern Cooperative Oncology Group performance status of 0–1. Patients should also fulfill the following biochemical and laboratory criteria based on tests performed within 6 months before enrollment: absolute neutrophil count, ≥ 1500 cells/mm^3^; platelet count, ≥ 50,000 cells/mm^3^; hemoglobin, ≥ 8.0 g/dl; normal kidney function within 6 months before enrollment as determined by creatinine < 2.0 ng/dl; normal liver function within 6 months before enrollment as determined by total bilirubin < 1.5 times the maximum normal value; alanine aminotransferase or aspartate aminotransferase < 2.5 times the maximum normal value.

### Exclusion criteria

Patients who fulfill the following criteria are excluded from this trial: distant metastasis; pelvic lymph node metastasis; history of ADT within 6 months before enrollment; history of definitive treatment for prostate cancer such as radical prostatectomy; history of pelvic irradiation; diagnosis of double primary cancer other than skin or thyroid cancer.

### Dropout criteria

Patients who desire to exit the trial or withdraw consent will be dropped out of the study. Additionally, any medical issues as well as unexpected serious complications occurring during treatment or the follow-up period that hinder completion of the study protocol will result in patient dropout.

### Treatment implementation

ADT will be initiated 3 months before WPRT (Fig. 1). In the intermediate-risk group, ADT will be administered for 6 months, whereas in high-risk group, long-term ADT for 2–3 years will be administered. Administration of antiandrogens and/or luteinizing hormone-releasing hormone agonists is allowed for patients in this study.

Before computed tomography (CT) for treatment planning, an experienced radiologist will insert three gold intra-prostatic fiducial markers via endorectal sonography for CyberKnife tracking. One week later, enhanced CT scans with a slice thickness of 2.5 mm will be obtained in all patients who will have empty bladders and rectal balloons in place. Participants should be in a relaxed supine position facilitated by an ankle pillow. Afterwards, a radiation oncologist will delineate organs-at-risk (OAR) and target volumes. If available, fusion with diagnostic magnetic resonance imaging (MRI) is recommended. Gross target volume (GTV) includes whole prostate glands, involved extraprostatic tissues, and any suspicion of involvement of the seminal vesicles. If seminal vesicle invasion is excluded definitely, they will not be included in the GTV calculation. Clinical target volume (CTV) includes regional nodal areas including obturator, external/internal iliac, and presacral lymphatic areas. Planning target volume (PTV) is expansion of CTV margins by 5 mm, except for the posterior 3-mm margin for rectal sparing. PTV must be irradiated with ≥ 95% of the prescription dose. OAR includes the rectum, bladder, and femoral heads, and all are contoured according to the Radiation Therapy Oncology Group guidelines [18]. IMRT will be used, and the dose regimen will be 44 Gy in 2.2-Gy fractions every weekday. Dose constraints are as follows: (1) bowel (small and large), 30% of the entire bowel volume must not receive more than 40 Gy; (2) rectum, 60% of the volume must receive ≤ 40 Gy; (3) bladder, 35% of the bladder volume must receive ≤ 45 Gy; (4) femoral heads, 15% of the femoral head volume must receive < 35 Gy. WPRT will be initiated within 10 days after the day of CT for treatment planning. For image guidance, daily cone-beam CT will be used. Patients will be asked to empty their bladder before each fraction, while there will be no routine bowel preparation.

For SBRT boost planning, another non-enhanced CT with 1.25-mm slices will be obtained at 1 week before the start of the boost therapy. Patients will be in a supine position with a vacuum cushion and an ankle pillow and with an empty bladder and a Foley catheter inserted for delineation of the urethra. The rectal balloon will not be used for the boost therapy. Boost volume is identical to the GTV of WPRT delineation, whereas the PTV margin is 3 mm in all directions. The urethra as well as the rectum and bladder are also included in the OAR. The CyberKnife robotic radiosurgery system (Accuray Incorporated, Sunnyvale, CA, USA) will be utilized for the SBRT boost, and the PTV must receive ≥ 80% and < 120% of the prescribed dose. OAR dose constraints are as follows: (1) rectum, < 85% of prescription dose; (2) bladder and urethra, < 100% of prescription dose. Prescription doses are 18 Gy and 21 Gy in three fractions, every other day. Tracking for fiducial markers will be used for image-guided radiotherapy. An enema will be performed for bowel preparation before every treatment. The whole radiotherapy period will not exceed 10 weeks.

### Data collection and follow-up period

**Fig. 2:**
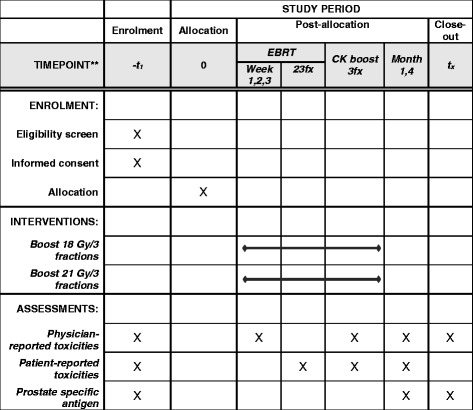
Intervention and assessment schedule for the trial according to the Recommendations for Interventional Trials (SPIRIT)

Patient data will be collected before start of radiotherapy, every week during WPRT, at the end of WPRT, at the end of SBRT boost, and at 1-month and 4-month follow-up visits after radiotherapy (Fig. 2). Patient data will be documented in Case Report Forms on urologic/gastrointestinal toxicities using the Common Toxicity Criteria for Adverse Events (CTCAE v 4.03), patient-reported outcomes (PRO), and PSA levels. After confirmation of the CyberKnife boost plan (at fourth week of radiotherapy), the evaluation of toxicities and efficacy will be no longer be blind. For ADT initiation, patients will be evaluated by the Department of Urology during follow-up visits every 3 months. After radiotherapy sessions, patients will also be evaluated by the Department of Radiation Oncology every 3 months for the first 2 years, every 6 months until 5 years, and annually thereafter. Follow-up evaluations will assess chronic toxicities and PSA levels. If clinical recurrence is suspicious, imaging studies, such as MRI and/or bone scans, will be considered.

For the patient data protection, separate identification code will be given to all patients. Also, data will be protected by password-accessible files, and only investigators can assess the files. All data will be deleted at 3 years after the end of the study.

### Assessment of primary and secondary endpoints

Primary endpoints of this trial are acute toxicities and short-term biochemical control. Acute toxicities are defined as events occurring within 3 months after treatment and are evaluated according to the CTCAE v 4.03 and PRO, using the Overactive Bladder Symptom Score and the International Prostate Symptom Score. Secondary endpoints are chronic toxicities and long-term biochemical control. Chronic toxicities are evaluated using the Late Effects of Normal Tissues scoring system. Biochemical recurrence (BCR) is defined as the Phoenix consensus definition of a rise in PSA level by 2 ng/ml or more above the nadir value [19].

The trial design and protocol adhere to the Recommendations for Interventional Trials (SPIRIT) criteria. The SPIRIT Checklist and Figure can be found in Additional file 1: Table S1 and Fig. 2.

### Statistical analysis

The present study is being performed as a purely exploratory pilot study; therefore, by calculation for statistical analysis, a total 15 patients in each treatment group is considered as an appropriate sample size. Comparison of the treatment two groups will be conducted using the chi-square test or Fisher’s exact test. Biochemical recurrence-free survival (BCRFS) will be calculated from the ADT start date using the Kaplan-Meier method. Descriptive statistics of all analyzed parameters will be provided, whenever appropriate.

## Discussion

A large comprehensive review involving over 52,000 patients reported that combination therapy including EBRT and brachytherapy appears superior to localized treatment alone in intermediate- and high-risk prostate cancer patients [6]. Additionally, Spratt et al. reported that compared with high-dose IMRT alone, brachytherapy in combination with IMRT was associated with better 7-year BCRFS rates (81.4% vs 92.0%, *P* < .001) and better distant metastasis-free survival (93.0 vs 97.2%, *P* = .04) for patients with intermediate-risk prostate cancer [4]. The BCRFS curve was gradually decreased until 10 years after IMRT alone, whereas there was no BCR incidence after approximately 7 years after the combination therapy. A recent Canadian phase III study demonstrated that patients receiving low-dose-rate brachytherapy boost were twice as likely to be free of BCR compared with those receiving dose-escalated EBRT boost [5]. They also reported that 5-, 7-, and 9-year BCRFS rates were 89%, 86%, and 83% with brachytherapy boost and 84%, 75%, and 62% with EBRT boost, respectively (*P* < .001). Similar to Spratt et al., the BCRFS curves for the treatment arms in the Canadian study sharply diverged after 4 years, and PSA control with brachytherapy boost reached a plateau after approximately 8 years compared with the continuous BCR events in the EBRT boost arm. Therefore, the advantage of combination therapy should increase with longer follow-up.

Despite better PSA control, one disadvantage of brachytherapy is the invasive procedure that requires anesthesia, pain control, and hospitalization. A dosimetric study found that CyberKnife could achieve the dose distribution of brachytherapy [7]. The difference of PTV receiving 100% of prescribed dose between CyberKnife and brachytherapy plans was measured only 0.5% (96.5% with CyberKnife vs 96.0% with brachytherapy). Furthermore, CyberKnife plans showed lower urethra doses and a rapid rectal dose fall off, suggesting a potential reduction in toxicities. More importantly, CyberKnife is non-invasive and controls intra-fractional movement through real-time fiducial tracking. Through the introduction of SBRT to boost therapy, overall treatment time can also be shortened to 5 weeks. SBRT itself has the potential to increase the therapeutic ratio as well, given that prostate cancer was reported to have a low α/β ratio with high sensitivity for large fraction sizes [8].

This trial uses PRO as well as CTCAE toxicity criteria for accurate assessment of toxicities. Although the CTCAE criteria are the “gold standard” for evaluating adverse events, attempts to capture patient perspective via PRO are increasing. A previous study found that agreement between the CTCAE criteria and PRO was poor to moderate [20]. Another study found that the number of patients reporting severe neuropathy (30%) was higher than that identified by the CTCAE criteria (10%), which suggested that toxicities were often undetected or underestimated by clinicians [21]. As a pilot trial, toxicity assessment is an essential endpoint, and this trial is anticipated to evaluate complications more precisely using PRO.

The goal of this prospective, single-center, pilot trial is to evaluate acute toxicities and short-term biochemical control after ADT and WPRT followed by SBRT boost. We attempt two boost dose schemes (18 Gy/3 fractions vs 21 Gy/3 fractions). We anticipate acceptable acute toxicities and favorable short-term control of PSA in intermediate- and high-risk prostate cancer patients who receive the combination treatment of ADT, WPRT, and SBRT boost. We will design the full-scale phase II study for selecting the boost dose referring to the data from the present pilot study.

## Trial status

Patient recruitment has not yet been completed.

## Additional file

Additional file 1: **Table S1.** SPIRIT 2013 Checklist. (DOCX 62 kb)

## Abbreviations

ADT: Androgen-deprivation therapy
BCR: Biochemical recurrence
BCRFS: Biochemical recurrence-free survival
BED: Biological effective dose
CT: Computed tomography
CTCAE: Common Toxicity Criteria for Adverse Events
CTV: Clinical target volume
EBRT: External-beam radiotherapy
GTV: Gross target volume
IMRT: Intensity-modulated radiotherapy
MRI: Magnetic resonance imaging
OAR: Organs-at-risk
PRO: Patient-reported outcomes
PSA: Prostate-specific antigen
PTV: Planning target volume
SBRT: Stereotactic body-radiotherapy
WPRT: Whole-pelvic radiotherapy
